# HIV risk, risk perception, and PrEP interest among adolescent girls and young women in Lilongwe, Malawi: operationalizing the PrEP cascade

**DOI:** 10.1002/jia2.25502

**Published:** 2020-06-30

**Authors:** Lauren M Hill, Bertha Maseko, Maganizo Chagomerana, Mina C Hosseinipour, Linda‐Gail Bekker, Audrey Pettifor, Nora E Rosenberg

**Affiliations:** ^1^ Department of Health Behavior University of North Carolina at Chapel Hill Chapel Hill NC USA; ^2^ Cecil G. Sheps Center for Health Services Research University of North Carolina at Chapel Hill Chapel Hill NC USA; ^3^ UNC Project‐Malawi Lilongwe Malawi; ^4^ Institute for Global Health and Infectious Diseases University of North Carolina at Chapel Hill Chapel Hill NC USA; ^5^ Desmond Tutu HIV Centre UCT Faculty of Health Sciences Cape Town South Africa; ^6^ Department of Epidemiology University of North Carolina at Chapel Hill Chapel Hill NC USA

**Keywords:** adolescent girls and young women, PrEP, cascade, HIV risk, Africa

## Abstract

**Introduction:**

As a user‐controlled HIV prevention method, oral pre‐exposure prophylaxis (PrEP) holds particular promise for adolescent girls and young women (AGYW). HIV prevention cascades, critical frameworks for the design and evaluation of PrEP programmes, outline the priorities of identifying individuals at greatest HIV risk and motivating them to initiate PrEP through perceived HIV risk. To inform future iterations of these cascades and PrEP delivery for AGYW, the objective of this study was to understand the level of interest in PrEP among AGYW at highest HIV risk, and the potential role of perceived risk in motivating PrEP interest.

**Methods:**

Using data from a cohort study of HIV‐negative AGYW in Lilongwe, Malawi (February 2016 to August 2017), we assessed the relationship between epidemiologic HIV risk (risk index developed in a previous analysis) and PrEP interest, and the extent to which perceived risk explains the relationship between HIV risk and PrEP interest. We further aimed to operationalize the pre‐initiation steps of the HIV prevention cascade in the study population.

**Results:**

In total, 825 AGYW were included in analyses, of which 43% met the criterion for high epidemiologic HIV risk. While epidemiologic risk scores were positively associated with PrEP interest, high numbers of AGYW both above and below the high‐risk cutoff were very interested in PrEP (68% vs. 63%). Perceived risk partially explained the relationship between HIV risk and PrEP interest; greater epidemiologic HIV risk was associated with high perceived risk, which was in turn associated with PrEP interest. Many more high‐risk AGYW were interested in PrEP (68%) than expressed a high level of perceived HIV risk (26%).

**Conclusions:**

These results highlight key relationships between epidemiologic HIV risk, risk perception and interest in PrEP. While risk perception did partially explain the relationship between epidemiologic risk and PrEP interest, there may be other important motivational mechanisms that are not captured in many HIV prevention cascades. The high number of participants with risk scores below the high‐risk cutoff who both expressed high perceived risk and interest in PrEP suggests that demand for PrEP among AGYW may not be well aligned with epidemiologic risk.

## 1 INTRODUCTION

Adolescent girls and young women (AGYW) in sub‐Saharan Africa are an important population for HIV prevention [1, 2]. In Malawi, AGYW ages 15 to 24 have more than twice the risk of HIV infection as their male counterparts [3, 4, 5]. This disproportionate burden of HIV risk among AGYW can be explained by a number of biological, social and behavioural factors [6], including difficulty negotiating condom use [7, 8]. To date, the predominant prevention technology available in Malawi has been the male condom, which is primarily male‐controlled and affords women limited ability to protect their sexual health. In contrast, oral pre‐exposure prophylaxis (PrEP) is an effective individually‐controlled prevention method, a promising alternative or additional prevention tool for AGYW [9, 10, 11].

Following World Health Organization (WHO) guidelines recommending offering PrEP to at‐risk populations, including AGYW in high burden settings [12], many countries including Malawi are beginning to make provisions to offer PrEP to AGYW. Despite the promise of PrEP and its impending rollout, we know little about the acceptability and potential uptake of PrEP among AGYW, including those in greatest need. With limited resources for PrEP [13], we need to understand if those AGYW at greatest HIV risk are the most likely to initiate PrEP. Furthermore, we need to understand *how* AGYW at greatest risk may become motivated to consider PrEP.

HIV prevention cascades provide valuable frameworks and metrics by which to evaluate the impact of prevention programmes, and to identify important intermediary endpoints to guide the development of effective prevention programmes [14]. The majority of these cascades begin by identifying the at‐risk population [14, 15, 16, 17], but the intermediary steps between identification of this population and initiation of a prevention method differ across cascades. That said, the thematic commonality between intermediary steps in many cascades are explicit or implicit mechanisms motivating initiation of a given prevention method. While Schaefer et al.’s 2019 unified cascade is the only to explicitly include a “motivation” step [14], and Hargreaves et al. similarly include “demand side” factors which may motivate or facilitate interest and access [18], the most commonly included motivational step is perceived HIV risk [15, 16, 18, 19]. The inclusion of risk perception in the majority of cascades implies that HIV risk perception is a key process through which individuals at elevated risk may be motivated to consider PrEP or other HIV prevention methods. However, empirical data on this implicit assumption are lacking. Understanding whether or not this mechanism is evident in AGYW can inform whether risk perception is an important target in programmes to promote PrEP interest and eventual uptake among high‐risk young women, and can be used to inform future iterations of the HIV prevention cascade to clarify priorities for motivational mechanisms to target.

To address this gap, using data from an observational cohort study with AGYW in Lilongwe, Malawi we aim to answer two primary questions: (1) Are those AGYW at highest HIV risk actually the most likely to (a) perceive themselves to be at risk and (b) express interest in PrEP use? and (2) To what extent does perceived risk explain the relationship between HIV risk and PrEP interest? We further aim to operationalize the pre‐initiation stages of the prevention cascade in the study population. The answers to these questions will build understanding of likely outcomes for key precursors to PrEP interest and uptake in the early days of rollout to AGYW.

## 2 METHODS

### 2.1 Study context

The Girl Power‐Malawi study was conducted at four health centres in Lilongwe, Malawi from February 2016 to August 2017 and assessed four service delivery models for AGYW [20, 21]. All clinics were in urban and periurban areas and had antenatal HIV prevalence levels of at least 5%. None of the models of service delivery included PrEP information or PrEP services. At the one‐year follow‐up participants were asked about their hypothetical interest in PrEP. The data presented here are taken from behavioural surveys from this trial.

### 2.2 Study participants and procedures

Two‐hundred and fifty AGYW were recruited from the catchment areas surrounding each of the four study clinics (n = 1000 total) through community outreach, participant referral, and self‐referral. AGYW were eligible to participate if they were 15 to 24 years old, from the catchment area, and willing to provide locator information (phone number and/or physical location). AGYW who were sexually active in the past six months were purposively recruited; study staff informally discussed romantic relationships and sexual activity with AGYW and invited those with current or past sexual activity for screening. Eligible and consenting participants were enrolled and followed for one year. All participants were asked to complete a behavioural survey at baseline, six months, and one year assessing socioeconomic, behavioural, biomedical and partnership characteristics; interest in using PrEP; and HIV risk perception. Surveys were administered in Chichewa by young female research officers using Open Data Kit software [22]. Phone and physical tracing were conducted for participants who missed research visits. Eight‐hundred and sixty‐seven AGYW participated in the one‐year visit (87% retention). The present analysis excludes AGYW who reported an HIV‐positive test result by the one‐year follow‐up visit (42 participants).

### 2.3 Ethical review

Girl Power‐Malawi received approval from the National Health Science Research Committee in Malawi and the University of North Carolina Institutional Review Board. Voluntary written informed consent was obtained from participants 18 to 24 years old. Assent and permission by a parent, guardian or authorized representative were obtained for adolescents 15 to 17 years old. In cases of limited literacy, an impartial witness was present.

### 2.4 Measures

#### 2.4.1 Epidemiologic HIV risk

Indicators of HIV risk used were previously identified as those associated with HIV incidence in the Girl Power‐Malawi cohort [23]. All nine identified risk factors were assessed at one year. Indicators included two sociodemographic factors (age 20 to 24; being separated/divorced/widowed), four sexual partnership characteristics in the past six months (≥2 partners; exchanging sex for money or gifts (transactional sex); having ≥1 partner ≥5 years older; and known/suspected partner concurrency), two sexually transmitted infections (STI) symptoms in the past six months (abnormal vaginal discharge; genital sores/ulcers) and having a previous pregnancy (measures previously described [24]). In our previous work to develop this risk index, AGYW with ≥3 risk factors were 15.2 times as likely to acquire HIV as those with <3 factors [23]. We summed the nine risk indicators by participant to create a risk score, and created a dichotomous of “high risk” indicator for some analyses (≥3 factors indicating “high risk”). This high‐risk cutoff was determined in the previous analysis; those with ≥3 risk factors had an HIV incidence rate >3 per 100 person‐years, the WHO high‐risk threshold [25].

#### 2.4.2 Perceived HIV risk

AGYW rated their perceived lifetime chance of acquiring HIV as: “no chance,” “small chance” or “high chance.” In bivariate analyses, this was dichotomized as “high chance” (1) versus other responses (0).

#### 2.4.3 PrEP interest

AGYW were rated their potential interest in using PrEP after receiving this explanation: “PrEP is a medicine that can be used to prevent HIV for people who are HIV‐negative. To be protected with PrEP, a pill is taken every day. These pills contain some of the same medicine used to treat people who already have HIV. PrEP is not currently available in Malawi.” Participants rated their interest in trying PrEP if it were available at the study clinic (“not at all interested,” “somewhat interested” or “very interested”). For multivariate analyses, this was dichotomized as “very interested” (1) versus other responses (0).

Control variables included highest level of education (grade level) and household economic status evaluated through an adapted Filmer Pritchett Wealth Index including 13 household assets [26]. A composite wealth score was created by weighting each asset by its factor loading on the first component in a principle components analysis, placing individuals on a continuous scale of relative wealth and categorizing scores into terciles [26].

### 2.5 Analysis

All analyses were performed in SAS v 9.4. We tested the following hypotheses: (1) Women with greater HIV risk will be more likely to a) be very interested in PrEP and b) perceive high HIV risk; (2) Women with greater perceived risk will be more likely to be very interested in PrEP; (3) Perceived risk will mediate the relationship between HIV risk and PrEP interest per hypotheses 1&2. We described the frequencies and percentages of categorical variables, and the median and inter‐quartile range of continuous variables (Table 1). We then estimated unadjusted and adjusted odds ratios of the association between each exposure and outcome of interest and corresponding 95% Wald chi‐square confidence intervals (Table 2). As adjustment for the control variables above did not qualitatively alter the results, only adjusted odds ratios are presented. To assess the assumption of linear associations between epidemiologic HIV risk and the response variables, we compared the fit of the linear model with three alternatives: a quadratic, a cubic, and a categorical model. Likelihood ratio tests did not indicate improved fit with these alternative models [27].

**Table 1:** Descriptive Statistics at one year (N = 825)

	n (%) or median [IQR]
Age	20 [18 to 22] (range: 15 to 27)
Highest education level completed	
Less than a primary school education	179 (21.8%)
Primary school completed (8 to 11)	417 (50.9%)
Secondary school completed	244 (27.3%)
Currently enrolled in school	351 (42.6%)
Household economic status	
Lowest	294 (35.6%)
Middle	239 (29.0%)
Highest	292 (35.4%)
Marital status	
Single	547 (66.3%)
Married	222 (26.9%)
Separated/Divorced/Widowed	55 (6.7%)
Sexually active in past six months	796 (96.5%)
PrEP interest	
Not all interested	99 (12.7%)
Somewhat interested	168 (21.5%)
Very interested	515 (65.9%)
Perceived lifetime HIV risk	
No chance	504 (61.1%)
Small chance	122 (14.8%)
High chance	149 (18.1%)
Do not know	49 (5.9%)
HIV risk	
Risk count score	
Risk factor count	2 [1 to 3] (range: 0 to 8)
High risk (≥3 risk factors of 9)	351 (42.6%)
Risk factors	
Age 20+	491 (56.6%)
Separated, divorced, or widowed	55 (6.7%)
≥2 partners in past year	178 (22.4%)
STI symptoms (past six months)	
Ulcerative symptoms	55 (6.7%)
Discharge symptoms	72 (8.8%)
Transactional sex	162 (18.8%)
≥1 partner suspected concurrent	390 (47.3%)
≥1 partner ≥5 years older than participant	227 (29.4%)
Ever pregnant	357 (43.3%)

**Table 2:** Associations between HIV risk factors, perceived HIV risk, and PrEP interest

Risk factors (ref = first)	PrEP interest (“very”)		Perceived “high” HIV risk	
	n (%)^a^	aOR (95% CI)	n (%)^a^	aOR (95% CI)
**Perceived HIV risk**				
No chance	295 (61.8%)	–	–	–
Small chance	83 (69.2%)	1.46 (0.95, 2.24)	–	–
High chance	111 (78.2%)	2.60 (1.68, 4.04)**	–	–
**Epidemiologic HIV risk**				
Risk factor count	–	1.13 (1.03, 1.24)**	–	1.28 (1.15, 1.42)***
Low/moderate risk (<3 risk factors)	297 (63.1%)	1.24 (0.92, 1.68)	58 (12.3%)	2.33 (1.60, 3.38)***
High risk (≥3 risk factors)	238 (67.8%)		91 (25.7%)	
**HIV risk indicators**				
Age 15 to 19	246 (74.1%)	0.65 (0.48, 0.89)**	60 (17.8%)	1.37 (0.94, 2.00)
Age 20+	269 (59.8%)		89 (20.4%)	
Not separated, divorced, or widowed	497 (64.6%)	1.24 (0.68, 2.27)	135 (17.5%)	1.32 (0.70, 2.53)
Separated, divorced, or widowed	38 (69.1%)		14 (25.5%)	
<2 partners in past year	369 (62.7%)	1.87 (1.27, 2.75)**	104 (17.7%)	1.50 (1.00, 2.27)
≥2 partners in past year	127 (76.1%)		43 (26.5%)	
STI symptoms (past six months)				
No ulcerative symptoms	475 (65.0%)	1.46 (0.78, 2.75)	136 (18.8%)	1.39 (0.72, 2.70)
Ulcerative symptoms	40 (78.4%)		13 (25.5%)	
No discharge symptoms	462 (65.3%)	1.14 (0.67, 1.92)	126 (17.9%)	1.97 (1.13, 3.42)*
Discharge symptoms	49 (71.0%)		21 (31.3%)	
No transactional sex	397 (62.7%)	2.24 (1.46, 3.44)**	117 (18.6%)	1.15 (0.73, 1.80)
Transactional sex	115 (78.8%)		32 (22.5%)	
No partner suspected concurrent	266 (61.2%)	1.42 (1.06, 1.90)*	49 (11.3%)	2.72 (1.86, 3.98)***
≥1 partner suspected concurrent	269 (69.0%)		100 (25.6%)	
No partner ≥5 years older than participant	416 (64.0%)	1.58 (1.13, 2.21)**	122 (18.9%)	1.55 (1.06, 2.27)*
≥1 partner ≥5 years older than participant	94 (77.1%)		26 (21.7%)	
Never pregnant	292 (67.4%)	0.99 (0.73, 1.35)	62 (14.1%)	1.73 (1.18, 2.54)**
Ever pregnant	222 (63.8%)		86 (25.8%)	

aORs adjusted for household economic status and education; aOR reference group is the first value listed in each row.

a Row percent reflecting proportion of respondents for each response category reporting being “very” interest in pre‐exposure prophylaxis (PrEP) (column 1), or a “high” perceived HIV risk (column 2).

b *p*<0.05;

c *p*<0.01;

d *p*<0.0001.

**Figure 1:**
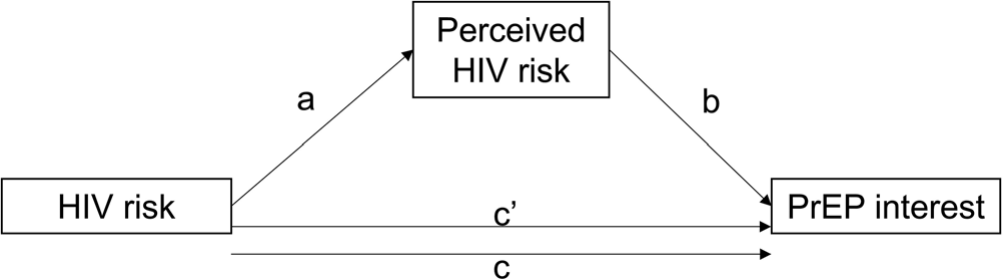
Mediation hypothesis and paths. PrEP, pre‐exposure prophylaxis

**Figure 2:**
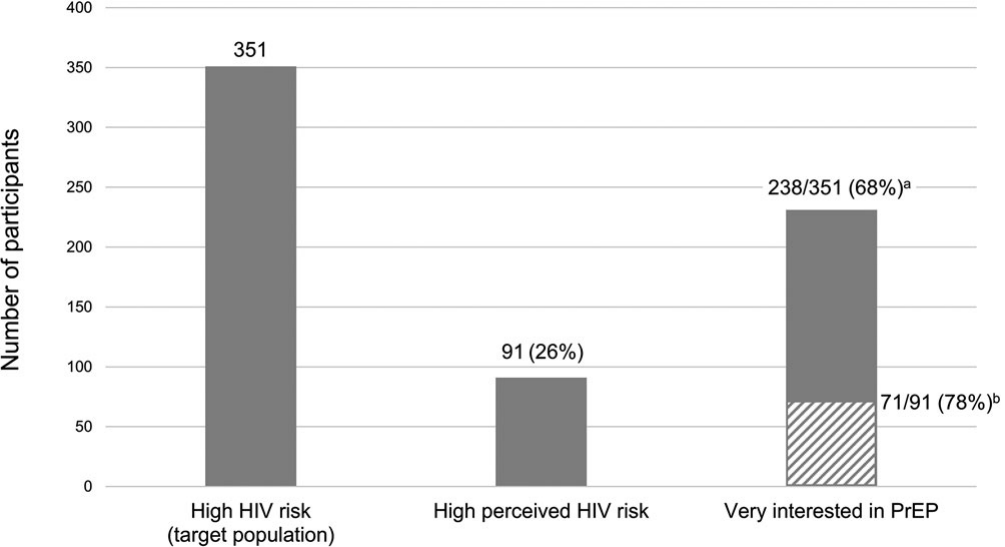
Operationalized pre‐initiation PrEP cascade. ^a^Proportion of high‐risk participants (n = 351) reporting being very interested in PrEP. ^b^Proportion of high‐risk participants with high perceived HIV risk (n = 91) reporting being very interested in PrEP. PrEP, pre‐exposure prophylaxis

We assessed the mediation hypothesis by estimating indirect and direct effects using the PROCESS macro v3.3 [28]. Statistical mediation was determined by assessing the statistical significance of the indirect effect (product of a*b; Figure 1) by the criterion of a non‐zero bootstrapped 95% confidence interval (5000 resamples) [28, 29]. Estimates for each path are adjusted for the control variables above; in this analysis we assume no unmeasured confounding for the causal effect of the mediator on the outcome. The proportion of the relationship between HIV risk and PrEP interest explained by the perceived risk mediator was calculated as 1‐c’/c (Figure 1) [28]. Finally, to characterize the “cascade” of level of perceived HIV risk and PrEP interest among high HIV risk AGYW, we calculated conditional frequencies of high perceived risk and being very interested in PrEP in this group (Figure 2). All hypothesis tests were completed using an imputed dataset. Given low levels of missing information (6% or less per variable), we employed deterministic imputation as follows [30]: Deterministic regression imputation for variables missing for >2% of participants; median imputation for variables missing for <2% of participants. PrEP, pre‐exposure prophylaxis; STI, sexually transmitted infections

## 3 RESULTS

### 3.1 Participant characteristics

In total, 825 AGYW completing the one‐year assessment not reporting an HIV‐positive test result were included. At one year, the median age of participants was 20 years old (Table 1). Half had completed primary school (51%) and a quarter (27%) had completed secondary school. The majority of participants (66%) were single. Nearly all participants reported being sexually active in the past six months (97%).

The median number of HIV risk factors reported was 2. 43% met the “high‐risk” cutoff of ≥3 risk factors. Most participants (61%) perceived no lifetime risk of HIV infection, while 15% and 18% perceived a small or high risk respectively. The majority (66%) were very interested in PrEP while 22% were somewhat interested, and 13% reported no interest.

### 3.2 Epidemiologic HIV risk and PrEP interest

The number of reported risk factors was associated with PrEP interest (Table 2); each additional risk factor was associated with 13% higher odds of being “very” interested in PrEP (aOR = 1.13; 95% CI: 1.03, 1.24). Slightly more AGYW reporting ≥3 risk factors (“high risk”) were very interested in PrEP, but this relationship was not statistically significant (Table 2). Four of the nine risk factors were associated with being very interested in PrEP in the expected direction: ≥2 partners, transactional sex, known/suspected partner concurrency, ≥1 partner ≥5 years older. Unexpectedly, women 20 years and older had *lower* odds of PrEP interest than those younger than 20 years. The other risk factors were not associated with PrEP interest.

### 3.3 Perceived HIV risk and PrEP interest

Both level of perceived risk and reporting “high” perceived HIV risk were associated with PrEP interest (Table 2). AGYW reporting high perceived risk had over twice the odds of being very interested in PrEP (aOR = 2.60; 95% CI: 1.68, 4.04) as those reporting lower perceived risk. PrEP interest was high among all perceived risk levels: 62% among women reporting no perceived risk, 69% among those with low perceived risk, and 78% among those with high perceived risk.

### 3.4 Mediation by perceived HIV risk: Indirect effect and direct effects

The indirect effect of epidemiologic HIV risk on PrEP interest through the mediator of perceived HIV risk was significant for both the binary indicator of high HIV risk and HIV risk score (Table 3; respective aORs: 1.14 95% CI: 1.05, 1.24; 1.04 95% CI: 1.01, 1.06). Perceived HIV risk explained 19% of the relationship between high epidemiologic risk and PrEP interest, and 25% of the relationship between HIV risk score and PrEP interest.

**Table 3:** Mediation results: indirect, direct and total effects of HIV risk on PrEP interest via perceived risk

	aOR (95% CI)	
	X = high HIV risk	X = HIV risk score
Indirect effect	1.14 (1.05, 1.24)	1.04 (1.01, 1.06)
Direct effect	1.19 (1.05, 1.24)	1.10 (1.00, 1.20)
Total effect	1.24 (0.92, 1.68)	1.13 (1.03, 1.23)
Proportion mediated	19.2%	25.2%

aORs adjusted for household economic status and education. PrEP, pre‐exposure prophylaxis; CI, confidence interval.

### 3.5 Operationalized pre‐initiation PrEP cascade

Many more high‐risk AGYW were interested in PrEP than perceived themselves to be at high risk (Figure 2); of the 351 high‐risk participants, only 26% perceived high HIV risk, while 68% were very interested in PrEP. Of the 91 high‐risk participants who also had high perceived HIV risk, 78% were very interested in PrEP (striped bar).

## 4 DISCUSSION

In summary, nearly half of AGYW reported risk factors placing them at high epidemiologic HIV risk. The majority expressed interest in PrEP, and epidemiologic risk was moderately associated with this interest. Only 26% of high‐risk participants reported high perceived risk, but nevertheless were more likely to perceive high risk than their lower‐risk counterparts. Perceived risk explained a quarter of the relationship between HIV risk and PrEP interest, suggesting that there may be other mechanisms motivating higher‐risk AGYW’s interest in PrEP.

### 4.1 HIV risk and PrEP interest

The majority of AGYW were very interested in PrEP, and this interest was moderately associated with higher HIV risk scores. Risk indicators most closely associated with PrEP interest included behavioural and partner factors which AGYW might recognize from HIV‐prevention education (e.g. partner concurrency, age disparate relationships). Approximately two‐thirds of both high‐risk and lower‐risk women were very interested in PrEP. This generally high level of PrEP interest has been found in other populations of AGYW and older women in South Africa [31, 32, 33] and Kenya [34]. Although these findings represent hypothetical interest, they suggest that demand for PrEP among AGYW may not be well aligned with epidemiologic HIV risk. Because of currently limited resources for PrEP in many settings, demand for PrEP according to HIV risk should be monitored to assess the need to better target PrEP delivery to women at greatest risk [35].

### 4.2 HIV risk and perceived risk

Although women with higher epidemiologic risk were more likely to report high perceived risk, 74% of those with high‐risk scores did not have high perceived risk. These results echo findings from FEM‐PrEP that half of women sero‐converting while taking PrEP had reported no perceived chance of HIV acquisition. This was attributed by the investigators to overestimation of protective behaviours and protective reasoning (e.g. minimizing perceived risk, cognitive avoidance) [36].

### 4.3 Perceived risk as a motivating mechanism

The mediation results indicated that perceived risk partially explained the relationship between epidemiologic risk and PrEP interest, thus there is reason to infer that perceived risk may be a motivating mechanism for PrEP interest in higher‐risk women. Future randomized studies should seek to understand if interventions promoting accurate risk perception could promote PrEP initiation [23]. Yet, perceived risk explained only a quarter of the relationship between risk scores and PrEP interest, indicating that there may be other unobserved motivators. Qualitative studies should seek to understand additional factors motivating AGYW’s PrEP interest beyond perceived risk. While some cascades include additional potential motivators, including attitudes towards the prevention method, social norms [18], and risk/benefit perceptions [19], more work is needed to understand the primary motivating mechanisms for PrEP initiation, to identify unified motivation indicators to inform and evaluate prevention programmes [14].

More research is needed to understand why many women who perceive no lifetime HIV risk would be very interested in PrEP. Previous evidence suggests that AGYW’s PrEP decision‐making can be driven by emotion and motivated reasoning about partner risk [37]. Our qualitative work with the study population suggests that fear of unplanned or uncontrollable risk factors including condom use errors, suspected/feared partner concurrency, and fear of rape were motivators for PrEP interest [38]. These factors could be seen as sources of HIV risk yet unlikely or hypothetical and therefore may not necessarily impute to a high perceived HIV risk while still serving to motivate interest in PrEP. Building a better understanding of these motivations will be important for the development and consolidation of PrEP‐specific and other HIV prevention cascades [15, 16, 19]. Inclusion of cascade indicators reflecting a more robust understanding motivators for interest in PrEP and other HIV prevention technologies will encourage activities to target the most important precursors to PrEP uptake and prevention‐effective use [14].

### 4.4 The cascade framework and PrEP

Unlike HIV treatment cascades, our operationalized pre‐initiation PrEP cascade (Figure 2) was non‐linear: many more AGYW with high risk scores were interested in PrEP than perceived themselves to be at high risk. There have been similar findings in men who have sex with men [39, 40]. This finding could be partially attributed to the fact that acknowledging interest in PrEP may present a lower “threshold” with regard to social desirability than acknowledging HIV risk behaviours or perceptions of HIV risk. This highlights a potential weakness of the cascade framework in defining progress towards PrEP initiation in high‐risk individuals. While each bar in an HIV treatment cascade is generally a subset of the previous one, this may not be the case for PrEP. This potential characteristic could make setting 90‐90‐90 type targets for PrEP difficult, as the denominator for each step may be difficult to define.

Our results highlight one further question about the application of the cascade framework for PrEP. Over 60% of participants with low/moderate‐risk scores were very interest in PrEP, and 12% of women with these scores reported high perceived risk. Should these women be excluded from the PrEP “target population” as defined by cascades? Women may be reticent to report risk behaviours [41, 42], or may fear risks that could not be reported as current, known risk factors, as those assessed in most common risk indices (as in our qualitative work [38]). Future studies should seek to understand the potential implications of more expansive definitions of the target population and PrEP eligibility criteria based on perceived risk and expressed PrEP interest. This evidence is needed to inform policy discussions to determine the potential societal costs and benefits of offering PrEP to those who may be highly motivated to use it but are not at highest assessable risk. Future studies should also seek to understand the downstream effects of HIV risk perception and other potential motivators of PrEP use on retention in care and adherence, as qualitative evidence suggests that low perceived risk and insufficient motivation for PrEP use may be related to the low levels of adherence among women in PrEP demonstration trials [43, 44, 45, 46, 47].

## 5 Limitations

The results of this study should be interpreted with key limitations in mind. First, participants’ reports of PrEP interest, sexual behaviours and perceptions of risk were likely susceptible to social desirability bias. To mitigate this issue, participants were assured that their responses were confidential and that there were no right or wrong answers. We also used the highest threshold (“very”) for PrEP interest to increase the theoretical specificity of this indicator. Second, few participants had previous knowledge of PrEP and PrEP was not available in the study setting at measurement, thus their reported interest in PrEP was hypothetical and based on limited consideration. Third, perceived HIV risk was measured with a single item, and thus may not capture all dimensions of perceived risk. Fourth, due to low cell counts for the associations between STI symptoms and PrEP interest, and between being separated/divorced/widowed and PrEP interest, tests may have been underpowered. Fifth, causal interpretations of all measures of association presented should be made with caution as they represent cross‐sectional associations; qualitative studies are needed to better understand the causality of the relationships studied. Finally, sexually active AGYW were purposively recruited for participation from urban and periurban settings in Lilongwe. The findings of this study are primarily generalizeable to sexually active AGYW living in similar urban and periurban settings in the region.

## 6 Conclusions

Our results bring to light relationships between epidemiologic risk, risk perception and PrEP interest and indicate directions for future research to inform effective HIV prevention programmes. A better understanding of mechanisms motivating PrEP interest beyond perceived risk is needed to inform delivery of PrEP among AGYW in high burden settings. Our results suggest that early demand for PrEP may not be well aligned with epidemiologic risk in this population; more research is needed to understand the implications of expanding or retaining current target population and PrEP eligibility definitions which may exclude many interested and motivated potential users not meeting a cutoff for epidemiologic risk.

## Competing interests

The authors have no competing interests to declare.

## Authors’ contributions

LH conducted the analysis and wrote the manuscript under the guidance of NR. NR conceptualized and led the Girl Power study. MH contributed to the overall study conceptualization and HIV‐related analyses. LB and AP Contributed to the overall study conceptualization and design. BM contributed to study design and study implementation. MC contributed to data analysis. All authors contributed to the writing of the manuscript and approved the final manuscript for publication.
